# Circadian‐rhythm‐regulating hormones: Key factors to regulate breast cancer metastasis via circulating tumor cells

**DOI:** 10.1002/mco2.189

**Published:** 2022-12-02

**Authors:** Chenguang Liu, Lingxiao Guo, Caiyun Fu

**Affiliations:** ^1^ Zhejiang Provincial Key Laboratory of Silkworm Bioreactor and Biomedicine Zhejiang Sci‐Tech University Hangzhou P. R. China; ^2^ College of Life Sciences and Medicine Zhejiang Sci‐Tech University Hangzhou P. R. China

1

Recently, a study in *Nature*, Diamantopoulou et al. presented breast cancer metastasizes faster during sleep in both mouse models and breast cancer patients. The circulating tumor cells (CTCs), as pioneers of the breast metastasis, were regulated by circadian‐rhythm‐regulating hormones in terms of both the level and activity in the blood (Figure 1).^1^

**FIGURE 1:**
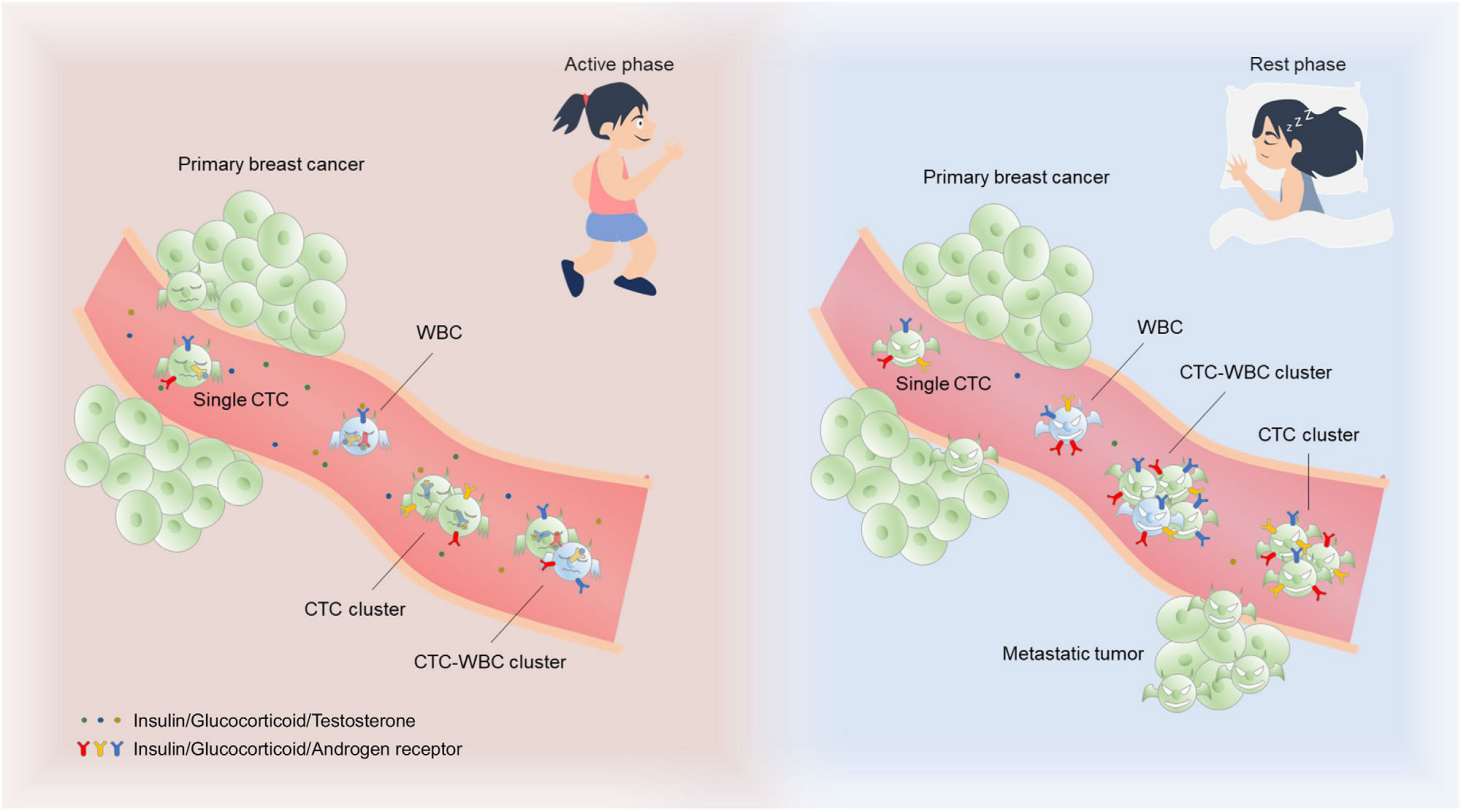
Breast cancer metastasis during active and rest phases. Breast cancer patients have higher levels and more metastatic ability of circulating tumor cells (CTCs) in their blood during the rest phase than the active phase due to the lower level of circadian‐rhythm‐regulated hormones, including insulin, glucocorticoid, and androgen

Among the many cancers, breast cancer is prone to metastasis. While, once metastasis occurs, treatment can become very tricky. Metastasis of breast cancer is mainly through the hematogenous dissemination of CTCs.^2^ However, current research has not focused much on when tumors shed cells and metastasize.

By collecting and analyzing blood samples from 30 female breast cancer patients (nine with stage IV metastatic breast cancer and 21 with early breast cancer and no metastasis) at different points in the day, the authors first found the levels of CTCs in blood samples collected during nighttime sleep (4 a.m.) is significantly higher than that collected in daytime activity (10 a.m.). To further verify the generality of the events, the authors constructed four different mouse models of breast cancer (i.e., an immunocompetent syngeneic breast cancer model, xenografts obtained from human breast CTCs, and two xenografts with established mouse breast cancer cells or human breast cancer cells, respectively). The microfluidic CTC capture and means of terminal blood sampling results were consistent with patient's events, where most CTCs in samples were achieved during the rest phase (all above 80%). Interestingly, when the light/dark cycle were changed to 14/10 h, 20/20 h, or 14/14 h, CTCs generation all showed high levels at rest time. These results validated the authors' view that CTCs tend to spontaneously intravasate during sleep.

Whereafter, Diamantopoulou et al. focused on the metastatic capability of CTCs during rest and active phases, respectively. The CTCs that shed spontaneously were collected from mice at different times in the day by microfluidics‐based capture. Subsequently, the captured CTCs were injected into tumor‐free recipient mice through the tail vein. The authors found that CTCs captured during the rest phase exhibited a remarkable metastasis ability compared to CTCs that were captured during the active phase. Following injection of fluorescence‐labeled CTC‐white blood cell (WBC) clusters, CTC clusters, and single CTCs, respectively, the tumor‐free recipient mice were detected with fluorescence distribution. Compared with mice with injection of single CTCs, mice injected with CTC‐WBC clusters and CTC clusters exhibited significantly high bioluminescence in their lungs. Thereby, through precise cell screening techniques, this study not only verified that CTCs generating during the rest phase had a more remarkable metastatic ability but also found the metastatic capability of CTC clusters was more vital than that of single CTCs.

Based on the above findings, it is reasonable to think of examining the difference of CTCs spontaneously shed at different times in gene expression. Following the single‐cell RNA sequencing, as expected, authors found CTCs isolated during the active and rest phase differed dramatically in gene expression. The mitosis and cell division pathways were highly engaged during the rest phase, while pathways that supported ribosomal biogenesis and gene translation were upregulated during the active phase. Consistent with the result of metastatic ability, the CTC cluster and CTC‐WBC cluster had significantly higher levels of gene upregulation than single CTC, which confirmed the conclusion that the metastasis‐forming properties of CTC cluster and CTC‐WBC cluster were greater than those of single CTCs from the genetic level. The same standard ways of gene expression and pathway activity were also observed in CTCs isolated from breast cancer patients at rest and active phase, respectively. And, the authors also observed this oscillatory proliferation timing in the primary tumors in a mouse model.

From a biochemical point of view, there must be substantial changes behind the circadian alternation to induce CTCs changes. In this case, the authors focused on the interstitial fluid pressure, interaction with immune cells and hemolysis‐rate‐induced damage. However, CTCs isolated during the rest and active phases did not differ significantly in these aspects. Subsequently, prompted by RNA‐seq data, the authors shifted their research to the expression of receptors for molecules, growth factors, and circadian‐rhythm‐regulated hormones. Fortunately, through extensive screening and comparison, they found that glucocorticoid, androgen, and insulin receptors were highly expressed in CTCs. By measuring tumor volumes and quantifying CTC abundance of tumor‐bearing mice treated with insulin and glucose, respectively (daily for 1 week during the rest phase), the authors found that daily oscillations of crucial circadian regulatory hormones can affect the proliferation and intravasation of breast cancer cells due to overexpression of their receptors. Furthermore, CTCs could be reduced by treatments of responsive ligand (e.g., dexamethasone, a specific ligand for glucocorticoid receptor, and testosterone, a main ligand for androgen receptor).

As we know, sleep is closely related to hormone regulation, protein damage, and repair and other physiological processes, thus affecting many diseases.^3^ With the revelation of circadian regulatory hormones regulating CTCs, the concern about sleep promoting breast cancer metastasis are relieved. The results means that we still have strategies to limit the number and activity of CTCs through moderate regulating hormone level or their receptors rather than shortening sleep time of patients with breast cancer.

In summary, in this work, the authors revealed the time‐dependent properties of breast cancer metastasis. They clarified the potential impacts of circadian‐rhythm‐regulating hormones in this metastatic process without using very high‐end equipment or extremely cutting‐edge technical tools. Notably, CTCs have been noticed in not only breast cancer but also liver cancer, lung cancer, colorectal cancer, etc.^4^ Although there is no evidence to prove whether the regulatory hormones affect other cancer metastasis, we believe that this research methodology can be extended to other cancer types. In addition, based on the mechanism that CTCs are regulated by circadian regulatory hormones, the rational design of the treatment time of conventional therapeutics may impact the treatment outcome. And on the premise of ensuring moderate sleep, the proper regulation of hormone level may also be a strategy to assist in controlling breast cancer metastasis. Considering that the mechanism of many therapeutics have been associated with blocking cell division,^5^ further studies of such time‐dependent gene‐expression changes in primary or metastatic tumors can more effectively optimize chemotherapy dosing habits in the clinic.

## AUTHOR CONTRIBUTIONS

Chenguang Liu and Lingxiao Guo conceived and drafted the manuscript. Chenguang Liu drew the figures. Caiyun Fu provided valuable discussion and revised the manuscript. All authors have read and approved the article.

## CONFLICT OF INTEREST

The authors declare that they have no conflict of interest.

## ETHICS STATEMENT

Not applicable.

## Data Availability

Not applicable.
